# A high risk of osteosarcoma in individuals who are homozygous for the p.D104N in endostatin

**DOI:** 10.1038/srep16392

**Published:** 2015-11-06

**Authors:** Wen-Zhi Bi, Dian-Wei Li, Song Luo, Zhi-Gang Song, Yun Wang, Hua Jin, Yan Wang, Qing Li, Meng-Xia Li, Dong Wang, Bo Sun, Meng Xu, Cheng-Xiong Xu

**Affiliations:** 1 Departments of Orthopaedics, The General Hospital of Chinese People’s Liberation Army, Beijing 100853, China; 2 Department of Pathology, The General Hospital of Chinese People’s Liberation Army, Beijing 100853, China; 3 Department of Molecular Pharmacology and Physiology, University of South Florida Health Sciences Center, Tampa, FL 33647, USA; 4 Cancer Center, Daping Hospital and Research Institute of Surgery, The Third Military Medical University, Chongqing 400042, China; 5 State Key Laboratory of Bioelectronics, School of Biological Science and Medical Engineering, Southeast University, Nanjing 210096, China

## Abstract

The D104N polymorphism (p.D104N) in endostatin has been previously identified in many types of cancer, and this polymorphism is believed to be a phenotypic modulator in some tumors. However, it is unknown whether endostatin p.D104N affects the risk and progression of osteosarcoma (OS). Here, we analyzed the p.D104N endostatin variant in 236 patients with OS and 418 healthy individuals. Similar frequencies of wild type and heterozygous p.104DN endostatin were observed in controls and OS patients. Interestingly, the frequency of the homozygous p.D104N (p.104NN) genotype was higher in OS patients group compared to control group, suggesting that individuals with p.104NN endostatin have a significantly increased risk for OS. In addition, OS patients with p.104NN endostatin had a shorter survival time and a higher rate of metastasis than OS patients with wild type endostatin. Animal experiments revealed that overexpression of p.104NN endostatin did not significantly inhibit OS lung metastasis. Interestingly, administration of endostatin dramatically inhibited OS lung metastasis in the p.104NN endostatin xenograft model. Together, these results suggest that p.104NN of endostatin is associated with the risk of OS and demonstrates predictive significance for clinical outcome in OS patients. In addition, endostatin therapy may be necessary for OS patients harboring p.104NN endostatin.

Osteosarcoma (OS) is the most common primary malignant tumor in children^1^, and 30% of children diagnosed with OS will not survive for more than 5 years^2^,^3^. Treatment of this disease often fails due to the development of metastasis^4^. However, the cellular mechanisms that underlie the development and metastasis of OS remain unclear.

Angiogenesis is a discrete event in carcinogenesis that is related to the aggressive potential of a tumor^5^,^6^. Accumulating evidence suggests that tumor growth is associated with increased angiogenesis and that the formation of new blood vessels is a fundamental step in tumor development and expansion^7^. In addition, studies have suggested that increased angiogenic properties within a tumor are associated with poorer clinical outcomes^8^. As is the case for numerous types of cancer, OS is dependent on angiogenesis^9^. Therefore, anti-angiogenic therapy has become a vital part of the arsenal against OS^10^,^11^.

Endostatin is a 183-amino-acid proteolytic fragment that is produced by the cleavage of the C-terminal non-collagenous domain (NC1) of human type XVIII collagen; endostatin is an efficient anti-angiogenic molecule^12^. As an angiogenic inhibitor, endostatin prevents tumor growth and expansion by controlling the formation of new blood vessels. *In vitro* and *in vivo* studies have shown that endostatin treatment can increase apoptosis and decrease microvessel density and metastasis in many tumors, including OS^10^,^11^,^13^. A single nucleotide polymorphism, c.4309G > A (p.D104N), has been identified in the endostatin domain of COL18A1; this polymorphism affects a site that is conserved in humans and mice^14^,^15^. A polymorphism can influence gene function^16^ and recent studies show that some polymorphism is strongly associated with the clinical significance in cancers^17^,^18^. So, the frequencies of endostatin p.D104N variant has been previously evaluated in healthy individuals and various diseases including cancers^19^,^20^. Iughetti *et al.* reported that the presence of the p.D104N variant results in a 2.5-fold increased risk of prostate cancer^21^. In addition, studies in breast cancer showed that endostatin p.D104N is associated with invasive breast cancer^15^, and patients carrying this polymorphism exhibited shorter progression-free survival and overall survival compared to those with wild type endostatin^22^. However, it is not known whether this polymorphism in endostatin affects the risk and progression of OS.

Therefore, we aimed to determine the frequency of the wild type, heterozygous p.D104N (p.104DN), and homozygous p.D104N (p.104NN) endostatin genotypes in patients with OS and to determine the influence of this polymorphism on OS risk, biological features and clinical outcomes.

## Results

### Distribution of the p.D104N endostatin variant in OS patients

First, we analyzed the distribution of the p.D104N endostatin variants in the healthy control and OS patient groups (Table 1). Of the 236 OS patients, 205 were normal homozygous (wild type) and 23 were heterozygous for the p.D104N. Of the 418 healthy individuals, 376 harbored the wild genotype of endostatin, 39 had the p.104DN genotype and 3 had the p.104NN genotype. We observed no significant differences in the frequencies of the wild type and p.104DN genotypes between the OS patient group and the healthy control group. However, we observed a higher frequency of individuals who were homozygous for p.D104N in the OS patient group (3.4%) than in the control group (0.7%) (Table 1).

### OS patients with the p.104NN genotype of endostatin have worse clinical outcomes

**Figure 1:**
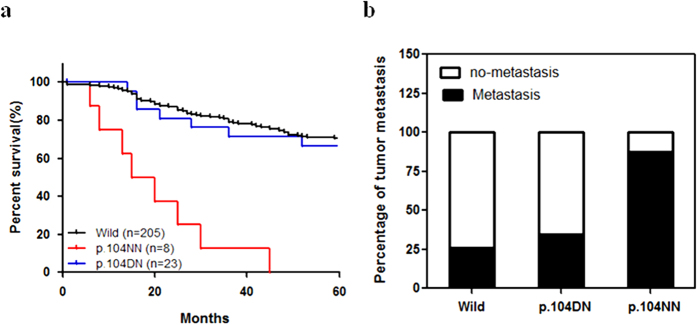
Homozygous D104N polymorphisms (p.104NN) in endostatin are associated with reduced survival and a high metastatic rate. (**a**) The survival rate was determined using the Kaplan-Meier method. The survival rate was significantly lower in osteosarcoma (OS) patients with p.104NN endostatin compared to OS patients who were heterozygous for the D104N polymorphism (p.104DN) or who harbored wild type endostatin. (**b**) The OS metastasis rate was higher in OS patients with p.104NN endostatin compared to OS patients with p.104DN or wild type endostatin.

**Figure 2:**
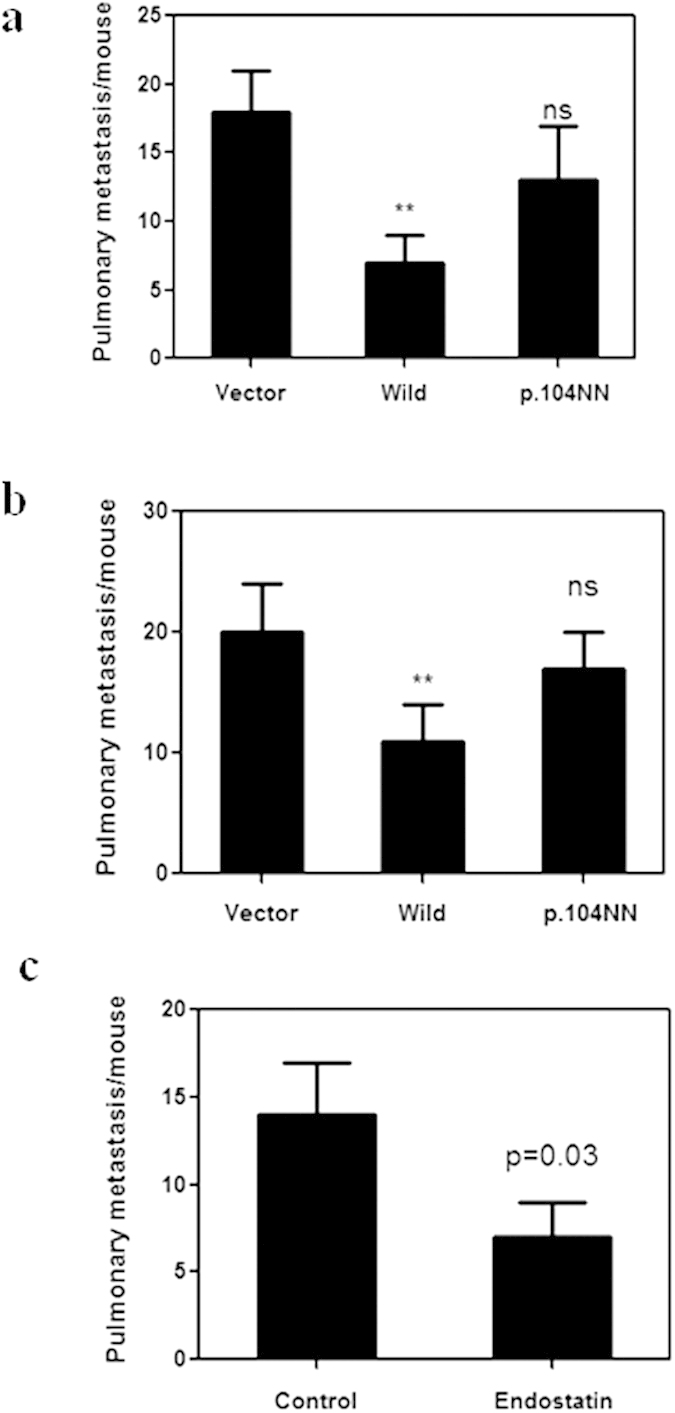
Overexpression of homozygous D104N (p.104NN) endostatin does not significantly inhibit osteosarcoma (OS) lung metastasis. (**a**) OS pulmonary metastasis was significantly inhibited by wild type endostatin but not by p.104NN endostatin (n = 8 mice). Mice were inoculated subcutaneously with LM8 cells that had been transfected with the indicated plasmid. Two weeks after inoculation, the mice were anesthetized, and the primary tumors were removed surgically. The lungs were removed 2 weeks after surgery, and the macroscopic pulmonary metastasis foci were counted. (**b**) Mice were inoculated subcutaneously with LM8 cells that had been transfected with the indicated plasmid. After the tumors reached 532 mm^3^ (±52), the mice were anesthetized, and the primary tumors were removed surgically. The lungs were removed 2 weeks after surgery, and the macroscopic pulmonary metastasis foci were counted. (**c**) Treatment with endostatin significantly inhibited OS lung metastasis in the p.104NN endostatin expressing LM8 xenograft model. Mice were inoculated subcutaneously with LM8 cells that had been transfected with the p.104NN endostatin plasmid. Two weeks after inoculation, the mice were anesthetized, and the primary tumors were removed surgically. Then, the mice were treated with endostatin (5 mg/kg, every 2 days) or PBS. The lungs were removed 2 weeks after surgery, and the macroscopic pulmonary metastasis foci were counted.

Next, we analyzed the association between p.D104N endostatin variants and clinical outcomes among OS patients. There was no difference in the clinical and laboratory features of OS patients with different p.D104N endostatin variants (Table 2). However, the OS patient group with the p.104NN genotype had a lower 5-year survival rate compared to the OS patient group with wild type or p.104DN endostatin (Fig. 1a). In addition, the OS patient group with the p.104NN genotype had a higher metastatic rate compared to the OS patient group with the wild type endostatin (Fig. 1b). In addition, we used an OS xenograft model to confirm the effect of p.104NN endostatin on OS lung metastasis. Similar to the clinical data, the animal experimental data revealed that overexpressing p.104NN endostatin did not significantly inhibit OS lung metastasis (Fig. 2a); wild type endostatin inhibits OS tumor growth^23^, and tumor size may be related to metastasis^24^. Therefore, in a subsequent experiment, we removed the primary tumors from animals with similarly sized tumors (532 ± 52 mm^3^) and counted the macroscopic pulmonary metastatic foci 2 weeks after surgery. Similar to the results of the previous animal study, p.104NN endostatin did not inhibit OS lung metastasis (Fig. 2b). Interestingly, treating the OS p.104NN endostatin xenograft model with endostatin significantly inhibited OS lung metastasis compared to control (Fig. 2c).

### The p.104NN endostatin genotype does not inhibit endostatin expression

**Figure 3:**
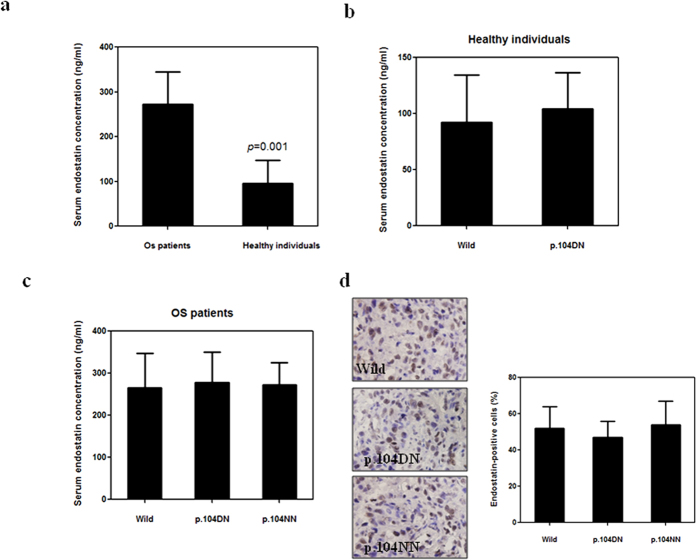
The D104N polymorphism (p.D104N) does not affect endostatin expression. (**a**) Serum endostatin levels were significantly higher in osteosarcoma (OS) patients (n = 236) compared to healthy individuals (n = 100). (**b**) The p.D104N does not affect serum endostatin levels in healthy individuals. (**c**) The p.D104N does not affect serum endostatin levels in OS patients. (**d**) The p.D104N does not affect endostatin expression in OS tissue. Immunohistochemistry assays were performed on primary tumor tissues from OS patients. Dark brown color indicates endostatin expression. Each bar represents the mean ± SD. Wild, wild type endostatin; p.104DN, heterozygous D104N polymorphism; p.104NN, homozygous D104N polymorphism.

Next, we investigated the effects of the p.104NN genotype on endostatin expression because a single polymorphism can influence gene expression^25^. In accordance with previous reports, the median serum endostatin value in the 236 OS patients (272.4 ng/ml) was higher than that in the 100 healthy individuals (95.1 ng/ml) (Fig. 3a). However, there was no difference in the median serum endostatin concentration between the 8 carriers of the wild genotype (95.7 ng/ml) and the 92 carriers of the p.104DN allele (94.5 ng/ml) among the healthy control individuals (*p* = 0.92; Fig. 3b). Similarly, there were no differences in the median serum endostatin concentration among the 8 carriers of p.104NN endostatin (272.9 ng/ml), the 23 carriers of p.104DN endostatin (278.7 ng/ml), and the 205 carriers of wild type endostatin (265.6 ng/ml; *p* = 0.79) within the OS patient group (Fig. 3c). Furthermore, consistent with these data, we did not observe any differences in endostatin expression in the tumor tissues from the OS patients with different p.D104N genotypes (Fig. 3d).

### The p.104NN genotype does not affect the anti-angiogenesis function of endostatin in OS

**Figure 4:**
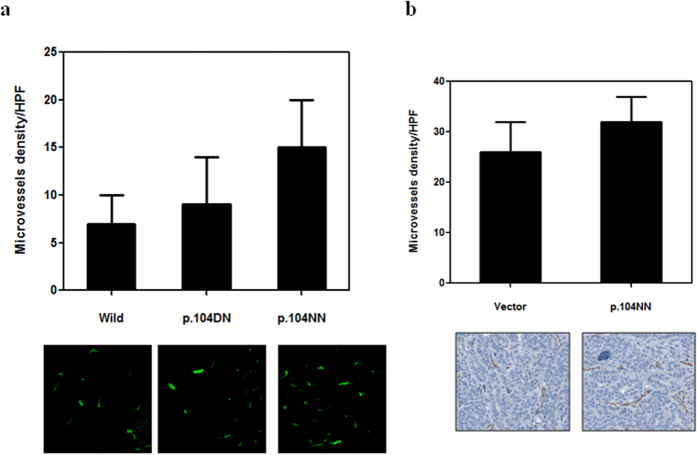
The D104N polymorphism in endostatin does not significantly affect angiogenesis in patients with osteosarcoma. (**a**) Microvessels were detected using CD31 immunofluorescence in primary tumor samples from OS patients. (**b**) Microvessels were detected in primary tumors from the LM8 xenograft model. Each bar represents the mean ± SD. Wild, wild type endostatin; p.104DN, heterozygous D104N polymorphism; p.104NN, homozygous of D104N polymorphism.

Endostatin inhibits tumor metastasis partly by inhibiting angiogenesis^26^, and p.D104N potentially impairs endostatin function^21^. Thus, we examined the effects of the p.104NN genotype on the anti-angiogenic activity of endostatin. First, we measured the microvessel density in tissue samples from OS patients with different endostatin p.D104N variants. Angiogenesis was slightly increased in OS patients with the p.104NN genotype, but the differences among these OS patients were not significant (Fig. 4a). In agreement with the clinical data, the animal experimental results showed that p.104NN did not significantly affect the anti-angiogenic activity of endostatin (Fig. 4b). Together, these data indicated that p.104NN does not affect the anti-angiogenesis function of endostatin.

## Discussion

The D104N polymorphism in endostatin has been previously reported in many types of cancer, and this polymorphism is believed to be a phenotypic modulator in some benign and malignant tumors^21^. However, these data are controversial, and different results have been reported in different cancer types. For example, studies have shown that the heterozygous p.D104N of endostatin is associated with an increased risk of prostate cancer^27^ and invasive breast cancer^15^ and with worse clinical outcomes for gastric cancer^22^, whereas no associations were observed between the heterozygous p.D104N genotype and the risk of multiple myeloma^28^ and lung cancer^29^. These discrepant results may be due to the heterogeneity of different cancers. This is the first study to report the frequency of the p.D104N polymorphism in endostatin in OS patients and to ascertain whether p.D104N alters the risk and clinical manifestations of OS.

In the present study, we observed no differences in the frequencies of the wild type and p.104DN genotypes of endostatin between healthy control individuals and OS patients. Interestingly, we found that individuals with p.104NN had a significantly greater risk of disease occurrence. These data suggest that p.104NN endostatin may be associated with OS susceptibility. In addition, our data revealed that patients with p.104NN endostatin have a shorter survival time and a higher rate of metastasis, suggesting that the p.104NN genotype of endostatin is associated with poor clinical outcome. Our animal experimental data demonstrated that overexpressing p.104NN endostatin does not significantly inhibit OS lung metastasis. Together, these data suggest that p.104NN endostatin may be a useful candidate marker for predicting OS disease progression.

Interestingly, the animal experiments showed that endostatin treatment can significantly inhibit OS lung metastasis in the p.104NN endostatin OS xenograft model, suggesting that endostatin therapy may be useful for preventing OS lung metastasis in OS patients with p.104NN endostatin. However, we did not observe a significant association between the different p.D104N endostatin variants and tumor aggressiveness in the OS patients. This result may be due to the inclusion of relatively few individuals with the p.104NN variant in this study, suggesting future study is needed in the lager OS patient population. A similar result has been observed for other tumor types. Lourenco *et al.* first reported that the p.104NN endostatin genotype is present in patients with sporadic breast cancer (SBC) but absent in control individuals, suggesting that the p.104NN genotype of endostatin is associated with SBC susceptibility. They also observed no association between the p.104NN endostatin genotype and tumor aggressiveness^30^, which is similar to the results reported here.

According to previous reports, polymorphisms can alter gene expression and function^27^. So, we hypothesized that the decreased anti-tumor effect of p.104NN endostatin may be due downregulated expression or loss of function of endostatin. However, our data showed that p.104NN does not affect endostatin expression. Our results are supported by previously published studies^15^. These data suggested that the effect of p.104NN on the anti-tumor activity of endostatin was not due to the inhibition of endostatin expression. Therefore, we examined the effects of p.104NN on the anti-angiogenic activity of endostatin. Endostatin is an anti-angiogenic factor, and studies have shown that the antitumor effects of endostatin are mediated partly by inhibiting angiogenesis^31^. Unfortunately, angiogenesis was slightly increased in patients with p.104NN endostatin compared to patients with wild type endostatin. Similar results were observed in the animal experiment, suggesting that the diminished anti-tumor effects of p.104NN endostatin are not due to modulation of anti-angiogenic activity. Thus, the mechanisms by which p.104NN weakens the anti-tumor effects of endostatin are unclear, and future studies are needed.

In summary, our study is the first to report preliminary evidence that the p.104NN genotype of endostatin is associated with OS susceptibility and poor clinical outcome and that it has predictive significance for the clinical outcome of OS patients. In addition, endostatin therapy may be necessary for OS patients with p.104NN endostatin.

## Methods

### Materials

EDTA-containing vacutainer tubes were obtained from BD vacutainer (Franklin Lake, NJ, USA). Genomic DNA isolation kits were purchased from Qiagen (Germantown, MD, USA). Endostatin human ELISA kits and primary antibodies against CD31 and endostatin were obtained from Abcam (Cambridge, MA, USA). Permount, Hematoxylin and secondary antibodies conjugated to HRP or FITC green were obtained from Sigma (St. Louis, MO, USA). DAB peroxidase substrate kits and DAPI-containing mounting media for immunofluorescence were purchased from Vector Labs (Burlingame, CA, USA). Endostatin was obtained from Shandong Xiangsheng Maidejin Biological Pharmaceutical Co.

### Human specimens

All the experimental methods were carried out in accordance with the approved guidelines. Blood and tumor samples were obtained from 236 OS patients primary tumors during diagnostic surgical biopsies (no metastasis). Blood samples were also obtained from 418 healthy individuals. The characteristics of the OS patients and the controls are summarized in Table 3. This research was approved by the Research Ethics Board of the General Hospital of the People’s Liberation Army. After describing the research study and the related procedures, written informed consent was obtained from the adult patients or from legally authorized representatives if the patients were minors.

### Genomic DNA amplification and genotyping

Genomic DNA was isolated from the blood samples (healthy individuals) and the diagnostic biopsy specimens (OS patients) using a Qiagen genomic DNA isolation kit according to the manufacturer’s instructions. Genotyping was performed as described by Balasubramanian *et al.* using the same primers^15^.

### Immunostaining assay

The tumor tissues were fixed in 10% neutral buffered formalin, embedded in paraffin, and sectioned at a thickness of 4 μm. The tissue sections were deparaffinized in xylene and then rehydrated via an alcohol gradient. Antigen retrieval was then performed using 10 mM citrate buffer (pH 6.0). For immunofluorescence (IF), the tissue sections were directly incubated in a solution of 3% bovine serum albumin (BSA) in PBS for 1 h at room temperature (RT). For immunohistochemistry (IHC), tissue sections were incubated in 3% hydrogen peroxide in methanol for 10 min and then washed with PBS. Then, the tissue sections were incubated in a 3% BSA solution. The slides were then incubated with a primary antibody overnight at 4 °C. The following day, the tissue sections were washed and incubated with the secondary HRP-conjugated (for IHC) or FITC-conjugated (for IF, in the dark) antibodies at RT. After a 1-h incubation, the slides were washed with PBS in the dark. For IF, a cover slip was affixed using mounting medium with DAPI. For IHC, the substrate color was developed using a DAB peroxidase substrate kit, and the sections were counterstained with hematoxylin. Next, cover slips were mounted using Permount. The number of CD31-positive vessels was counted in four randomly selected 1 mm^2^ areas in a high power field (HPF), and the average number was calculated. To quantify the total surface area of the vasculature, the total perimeter of the vessels was measured in five randomly selected 0.25 mm^2^ areas using an image analyzer. Endostatin-positive staining was determined by counting 5 randomly chosen fields per section, and the percentage of DAB-positive cells per 100 cells was determined at ×400.

### ELISA assays

Blood samples were collected in EDTA-containing tubes, and the serum samples were separated and stored at −70 °C for future use. Serum endostatin levels were measured using a commercially available ELISA according to the manufacturer’s instructions. All of the measurements were performed in duplicate to ensure the accuracy of the collected data.

### Animal study

An animal study was performed as described by Kaya *et al.*^23^. Briefly, female BALB/c nu/nu mice (8 mice/group) were inoculated subcutaneously with LM8 cells that had been transfected with the indicated endostatin variant (1 × 10^6^ cells/mouse). One week after inoculation, blood samples were collected from the tail vein and analyzed for endostatin. At the indicated times after inoculation, the mice were anesthetized, and the primary tumors were removed surgically. The lungs were removed 2 weeks after surgery, and the macroscopic pulmonary metastatic foci were counted.

For the endostatin therapy experiment, the mice were inoculated subcutaneously with p.104NN endostatin-overexpressing LM8 cells (1 × 10^6^ cells/mouse). Two weeks after inoculation, the mice were anesthetized, and the primary tumors were removed surgically. After the primary tumor was removed, endostatin was administered to the treatment group by intraperitoneal injection (5 mg/kg, every 2 days). The control group received physiological saline. The lungs were removed 2 weeks after endostatin treatment, and the macroscopic pulmonary metastatic foci were counted. The experimental animal protocol was approved by the institutional review board of the General Hospital of the People’s Liberation Army and was performed in accordance with accepted guidelines for animal research.

### Statistical analyses

Hardy-Weinberg equilibrium was evaluated using the X^2^ statistic for the goodness-to-fit (using one degree of freedom). The statistical significance of the differences between groups was calculated using the X^2^ test or Fisher’s exact test. All the analyses were performed using the SAS statistical package for Windows, version 8.1 (SAS Institute Incorporation, USA).

## Additional Information

**How to cite this article**: Bi, W.-Z. *et al.* A high risk of osteosarcoma in individuals who are homozygous for the p.D104N in endostatin. *Sci. Rep.* **5**, 16392; doi: 10.1038/srep16392 (2015).

## Figures and Tables

**Table 1:** Endostatin genotype distribution among osteosarcoma patients and controls.

Genotypes	Controls number (%)	Patients Number (%)
p.104NN	3 (0.7)	8 (3.4)
p.104DN	39 (9.3)	23 (9.7)
Wild	376 (90.0)	205 (86.9)

Wild, no polymorphism; p.104DN, heterozygous polymorphism; p.104NN, homozygous polymorphism.

**Table 2:** Endostatin genotype distributions in osteosarcoma patients stratified by clinical and laboratory variables.

	Patients number	Wild+p.104DN(%)	p.104NN(%)	*p*-value
Sex				1.00
Male	139	134(96.4)	5(3.6)	
Female	97	94(96.9)	3(3.1)	
Age				1.00
20≤	162	157(96.9)	5(3.1)	
>20	74	71(95.9)	3(4.1)	
Histologic subtype				0.65
Osteoblastic	146	142(97.3)	4(2.7)	
Chondroblastic	35	33(94.3)	2(5.7)	
Fibroblastic	21	20(95.2)	1(4.8)	
Other	34	33(97.1)	1(2.9)	
Grade				1.00
III	61	59(96.7)	2(3.3)	
IV	175	169(96.6)	6(3.4)	
Enneking stage				0.72
2A	59	56(94.9)	3(5.1)	
2B	177	172(97.2)	5(2.8)	

Wild, no polymorphism; p.104DN, heterozygous polymorphism; p.104NN, homozygous polymorphism.

**Table 3:** Characteristics of osteosarcoma patients and controls.

	Patients number	Healthy individuals number	*p*-value
Sex			0.33
Male	139 (58.9)	229(54.8)	
Female	97(41.1)	189(45.2)	
Histologic subtype			
Osteoblastic	146(61.9)	NA	
Chondroblastic	35(14.8)	NA	
Fibroblastic	21(8.9)	NA	
Other	34(14.4)	NA	
Grade			
III	61(25.8)	NA	
IV	175(74.2)	NA	
Enneking stage			
2A	59(25.0)	NA	
2B	177(75.0)	NA	
